# Connection of BANK1, Tolerance, Regulatory B cells, and Apoptosis: Perspectives of a Reductionist Investigation

**DOI:** 10.3389/fimmu.2021.589786

**Published:** 2021-03-18

**Authors:** Ludmilla Le Berre, Mélanie Chesneau, Richard Danger, Florian Dubois, Damien Chaussabel, Mathieu Garand, Sophie Brouard

**Affiliations:** ^1^ CHU Nantes, Université de Nantes, Inserm, Centre de Recherche en Transplantation et Immunologie, UMR 1064, ITUN, Nantes, France; ^2^ Systems Biology and Immunology, Sidra Medicine, Doha, Qatar

**Keywords:** tolerance, transplantation, regulatory B cells, B cells, BANK1, apoptosis

## Abstract

BANK1 transcript is upregulated in whole blood after kidney transplantation in tolerant patients. In comparison to patients with rejection, tolerant patients display higher level of regulatory B cells (Bregs) expressing granzyme B (GZMB^+^) that have the capability to prevent effector T cells proliferation. However, BANK1 was found to be decreased in these GZMB^+^ Bregs. In this article, we investigated seven different transcriptomic studies and mined the literature in order to make link between BANK1, tolerance and Bregs. As for GZMB^+^ Bregs, we found that BANK1 was decreased in other subtypes of Bregs, including IL10^+^ and CD24^hi^CD38^hi^ transitional regulatory B cells, along with BANK1 was down-regulated in activated/differentiated B cells, as in CD40-activated B cells, in leukemia and plasma cells. Following a reductionist approach, biological concepts were extracted from BANK1 literature and allowed us to infer association between BANK1 and immune signaling pathways, as STAT1, FcγRIIB, TNFAIP3, TRAF6, and TLR7. Based on B cell signaling literature and expression data, we proposed a role of BANK1 in B cells of tolerant patients that involved BCR, IP3R, and PLCG2, and a link with the apoptosis pathways. We confronted these data with our experiments on apoptosis in total B cells and Bregs, and this suggests different involvement for BANK1 in these two cells. Finally, we put in perspective our own data with other published data to hypothesize two different roles for BANK1 in B cells and in Bregs.

## Background

We have previously reported that patients with a state of clinical operational tolerance after renal transplantation (TOL) (1) displayed T and B lymphocyte populations of specific phenotypes (2, 3). Tolerant patients showed reduced costimulatory signaling, immune quiescence, apoptosis, memory T cell responses, but a strong B cell transcriptional activity (4). This phenotype was associated with a higher number of peripheral B cells (3, 5–7) and an increase in BANK1 (B-cell scaffold with ankyrin repeats 1) gene expression (5) compared to patients with chronic rejection (CR), patients having stable renal function under immunosuppression (STA), and healthy volunteers (HV). In parallel, we previously reported that tolerant patients exhibit a higher number of IL21-dependent GZMB^+^ B cells that can inhibit effector T cell response by contact and through a GZMB-dependent manner (8). These GZMB^+^ B cells have a high capacity to proliferate under GZMB stimulation (9) and a higher sensitivity to apoptosis (10). Using bioinformatics and data-mining approaches to analyze key genes interconnected in blood from transplanted patients, we highlighted BANK1 gene as a key leader gene expressed in renal tolerance (11). Nonetheless, the molecular role of BANK1 in immune tolerance, especially in B cells subtypes, is still unknown. The objective of the present study was to explore publicly available transcriptomic data to decipher the molecular links between BANK1, tolerance, and regulatory B cell populations in health and diseases, and put in perspective our own data set and results, to highlight the putative role of BANK1 in post-transplantation tolerance.

## Reasoning Path

### Healthy Regulatory B Cells Constitutively Express Lower Levels of BANK1 Transcripts

**Figure 1:**
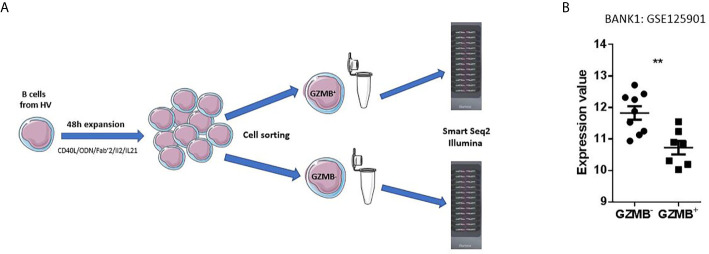
Primary observation. **(A)** Design of the primary dataset. A dataset deposited by our team in the NCBI GEO public repository (GSE125901) was used as starting point for selection of a gene candidate. This study was conducted in France on adult healthy subjects. B cells were sorted by magnetic sorting (Negative selection, Miltenyi, Human B cell isolation kit II) then expanded during 48 h in a stimulation medium composed of CD40L, ODN, Fab’2, IL2, and IL21. GZMB^+^ and GZMB^-^ B cells were separated by BD ARIA Cell Sorter and lysed. RNA was extracted to be subsequently profiled on Illumina HT12 Beadarrays. **(B)** Expression profile of BANK1 in GZMB^+^ and GZMB^-^ B cells obtained from publicly available data set GSE125901; ** denotes statistically significance difference with p < 0.01.

B cells from nine healthy volunteers (HV) were sorted by magnetic sorting (Negative selection, Miltenyi B cell isolation kit II) and expanded with a stimulation medium composed of CD40L, CpG ODN, Fab’2, IL21, and IL2 (12). Three-day post expansion, GZMB^+^ and GZMB^-^ B cells were isolated by flow cytometry (ARIA sorter, BD) and a >95% purity was obtained (data not shown). RNA was extracted from the lysed cells and transcriptomic profiles generated using Illumina Bead-arrays (**Figure 1A**). The RNA-Seq data are available in GEO under accession GSE125901 (**Table 1**).

**Table 1:** List of validation data sets, group’s information, changes in BANK1 expression, t test and F test.

GSE ID	Title	Group A	Group B	Exp A	Exp B	B/A	T-test	F-Test
GSE125901	Granzyme B-secreting B cells as a potential cell therapeutic target	GZMB- B cells (n=9)	GZMB+ B cells (n=7)	11.827	10.726	0.907	<0,005	<0,01
GSE76272	Transcriptomic Signature of the CD24hi CD38hi Transitional B Cells Associated With an Immunoregulatory Phenotype in Renal Transplant Recipients.	B cell (CD24^+^CD38^-^ and CD24^int^CD38^int^B cells) (n=10)	Breg (CD24^hi^CD38^hi^ B cells) (n=5)	8.877	8.383	0.944	<0,05	<0,05
GSE35002	Human regulatory B cells combine phenotypic and genetic hallmarks with a distinct differentiation fate.	B cell (IL10^-^B cells) (n=6)	Breg (IL10^+^ B cells) (n=6)	10.961	10.328	0.942	<0,001	<0,001
GSE50895	Gene expression profiling of human IL-10+ B cells	B cell (IL10^-^B cells) (n=5)	Breg (IL10^+^ B cells) (n=5)	10.799	10.423	0.965	<0,05	<0,05
GSE54017	CD40-activation of human B cells	resting B cells (n=4)	CD40 activated B cells (n=4)	855.693	354.100	0.414	<0,001	<0,001
GSE22529	Chronic lymphocytic leukemia: peripheral blood B cells (HG-U133B)	CLL B cells (n=41)	Healthy B cells (n=10)	7.648	9.914	0.771	<0,001	<0,001
GSE6691	Waldenstrom's macroglobulinemia: B lymphocytes and plasma cells	B cells from HV (n=8)	Plasma cells from HV (n=5)	10.250	4.779	0.466	<0,001	<0,001

Green color means <0.05, blue <0.01, orange <0.005 and red <0.001.

**Figure 2:**
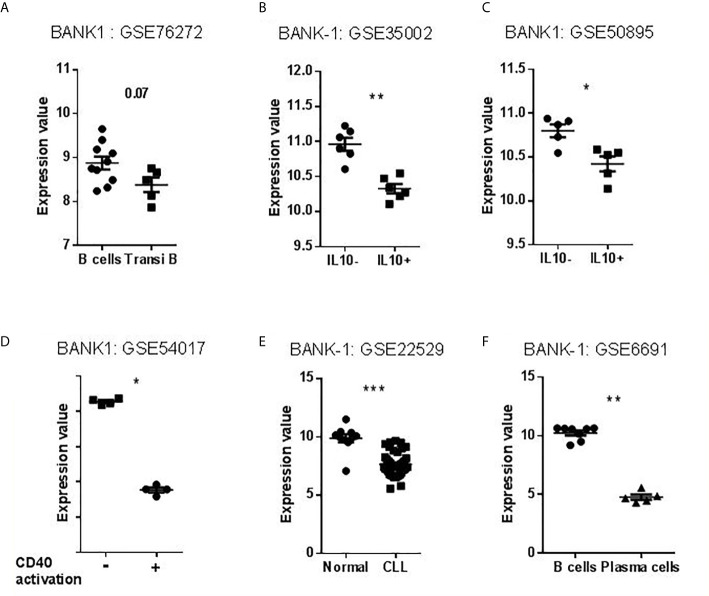
Transcriptional profiles of BANK1 in datasets selected for validation. **(A–C)** Levels of BANK1 expression in B cells compared to Bregs (CD24^hi^ CD38^hi^ Transitional B Cells or IL10^+^ B cells). **(D)** Levels of BANK1 expression in CD40 activated compared to resting healthy B cells (GSE54017). **(E)** Normal B cells (HV) compared to CCL B cells (GSE22529). **(F)** B cells compared to plasma cells from HV (GSE6691) (*p < 0.05, **p < 0.01, ***p < 0.001).

As seen in **Figure 1B**, we found BANK1 transcript to be significantly down-regulated in GZMB^+^ B cells compared with GZMB^-^ B cells (t test p < 0.005; F test < 0.01). Down-regulation of BANK1 was further observed in three independent public datasets available on GEO (**Table 1**). Bigot et al. (GSE76272) compared transitional B cells (CD24^hi^CD38^hi^ Bregs) with naive and memory B cells (CD24^+^CD38^-^ and CD24^int^CD38^int^) from healthy individuals (13) and observed a non-significant decrease in BANK1 expression in the CD24^hi^CD38^hi^Breg cells [**Figure 2A**; see (13) for Breg subtypes]. Both Van de Veen et al. and Lin et al. compared healthy human IL10^+^ Bregs with IL10^-^ B cells and found that BANK1 transcript levels were significantly decreased in IL10^+^ Bregs (GSE35002, p < 0.001 and GSE50895, p < 0.05) (**Figures 2B, C** **) (** 14, 15). These observations suggest that in health, BANK1 expression are lower among the different subtypes of regulatory B cells compared with non-regulatory B cells, suggesting perhaps some common functional properties, despite different phenotypes.

### Activated/Differentiated B Cells Exhibit Lower Levels of BANK1 Transcripts

To explore BANK1 expression in activated and differentiated B cells, we examined three additional public datasets: human resting B cells vs. CD40 activated B cells [GSE54017 (16)], human resting B cells vs. Chronic Lymphocytic Leukemia (CLL) patient B cells [GSE22529 (17)], and human resting B cells vs. plasma cells [GSE6691 (18)] (**Table 1**). We found that BANK1 expression is down-regulated, after CD40 activation (GSE54017, p < 0.001), in B cells from CLL patients (GSE22529, p < 0.001), as well as in plasma cells (GSE6691, p < 0.001) (**Figures 2D–F**). These three observations indicate that B cells with an activated/differentiated profile express lower levels of BANK1 transcripts compared to human resting B cells.

### Knowledge Gap Exists for Molecular Links Between BANK1, Tolerance, Transplantation, and B Cell Regulation

**Figure 3:**
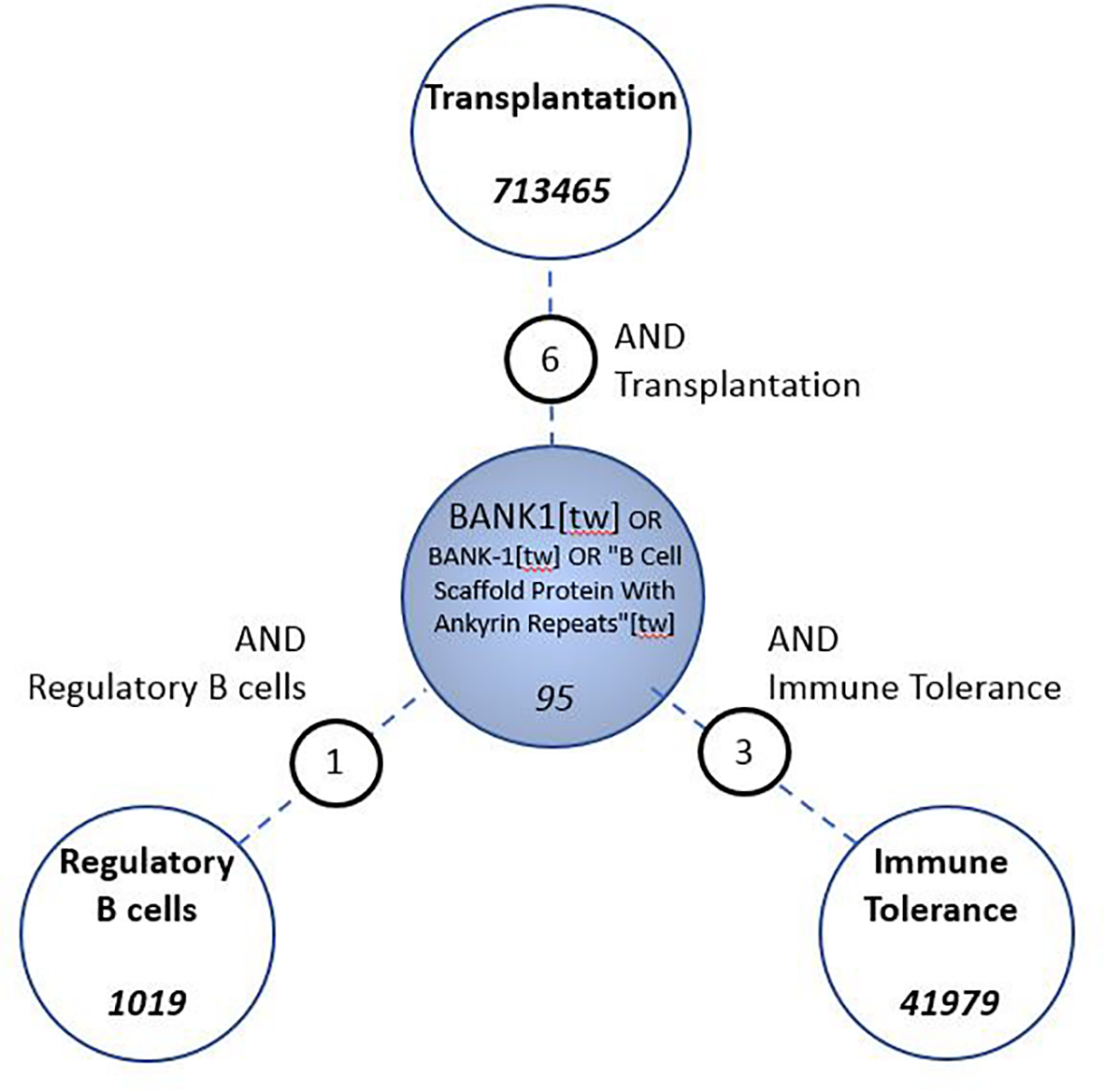
Assessment of gap in knowledge in the biomedical literature. PubMed searches were conceived to identify associations between BANK1 and Transplantation, Immune Tolerance and Regulatory B cells.

Since we first described BANK1 to be differentially expressed in blood from tolerant recipients, we investigated the current literature on the following terms: transplantation, immune tolerance and regulatory B cells which returned 713,465, 41,979, and 1,019 PubMed entries, respectively. In parallel, we queried PubMed using on the official symbol, name, and known aliases of our gene of interest (“BANK1” [tw] OR “B-cell scaffold with ankyrin repeats 1”[tw]) which returned 95 articles (as of Nov-2020). We identified only six, three, and one overlapping publications between BANK1 and “transplantation”, “immune tolerance”, and “regulatory B cells”, respectively (**Figure 3**). The low number of overlaps indicates a substantial knowledge gap in the current literature linking BANK1 to these topics.

### STAT1, FcγRIIB, TNFAIP3, TRAF6, and TLR7 Molecules Link Indirectly BANK1 to Bregs, Transplantation and Immune Tolerance

To further explore the current knowledge about BANK1’s role and its association with biological concepts and processes, a PubMed query restricted to title (i.e. [ti] instead of [tw] previously) was constructed. The search returned 42 articles (as of Nov 2020). From the title of the 42 articles, we manually extracted keywords and concepts which were grouped according to the following general categories: Auto-immune diseases, Other diseases, Cell types, Proteins, and Functions (**Table 2** **)**. Studies mentioning BANK1 highlighted the association with B cells and B cell function above other cell types. BANK1 was mainly associated with autoimmune diseases especially with systemic lupus erythematosus (SLE). In addition, literature associations with other proteins were identified, such as B lymphoid kinase (BLK), which is important for B cell receptor signaling and development. Other important immune-related proteins included IRF5, STAT4 and many members of the TNFR family (**Table 2**).

**Table 2:** Concepts extracted from the BANK1 literature and the prevalence of each terms.

Category	BANK1-associated Concept	PubMed articles
AI Disease	SLE	15
AI Disease	RA	4
AI Disease	Systemic sclerosis	3
AI Disease	Type 1 diabetes	1
AI Disease	Automimmune thyroid diseases	1
Other Disease	Neuropathy	1
Other Disease	LDL chlesterol	1
Other Disease	B-cell lymphoma	1
Other Disease	Polymyositis/dermatomyositis	1
Other Disease	Psoriasis	1
Other Disease	Primary Sjögren's syndrome	1
Cell Type	B cells	7
Cell Type	Regulatory T cells	1
Protein	BLK	12
Protein	IRF5	2
Protein	STAT4	2
Protein	STAT1	1
Protein	ATG5	1
Protein	ATG7	1
Protein	PTPN22	1
Protein	PTPN6	1
Protein	TNFAIP3	1
Protein	TNFSF4	1
Protein	TNFRSF14	1
Protein	TRAF6	1
Protein	IL-6	1
Protein	NF-kappaB	1
Protein	TLR7	1
Protein	p38	1
Protein	MNK1/2	1
Protein	eIF4E	1
Protein	PLCG2	1
Protein	CLEC2D	1
Protein	MyD88	1
Function	B cell signaling	2
Function	Antibody development	3
Function	Alters B cell responses	1
Function	Cytokine	1
Function	Apoptosis	1
Function	Innate immune sihnaling	1
Function	Tumor suppressor role	1

In gray, occurrences superior to 1 article. AI disease, autoimmune disease; SLE, systemic lupus erythematosus; SSc, systemic sclerosis; RA, rheumatoid arthritis.

**Figure 4:**
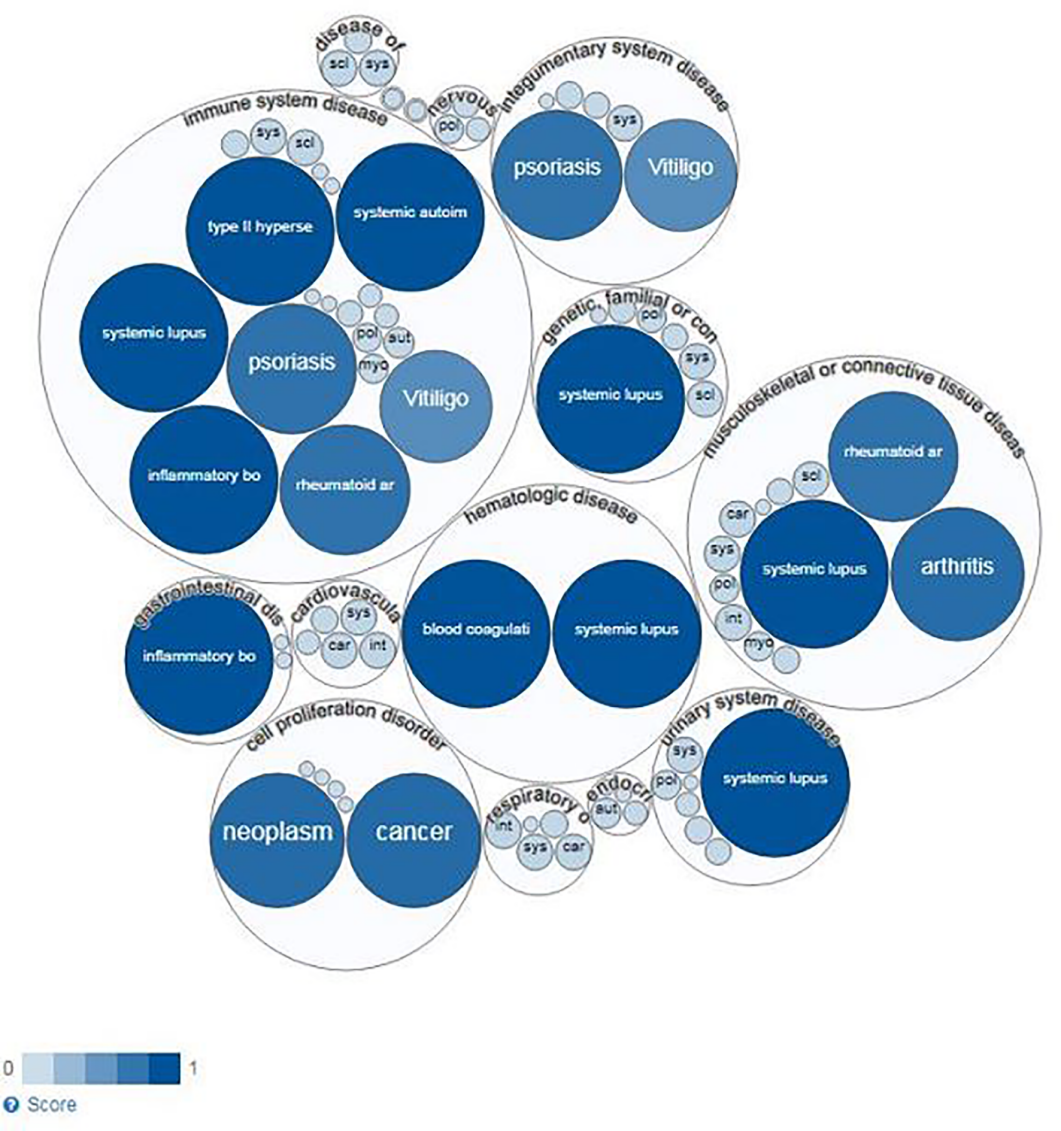
Diseases linked to BANK1 (Open Target Source) by text mining.

**Figure 5:**
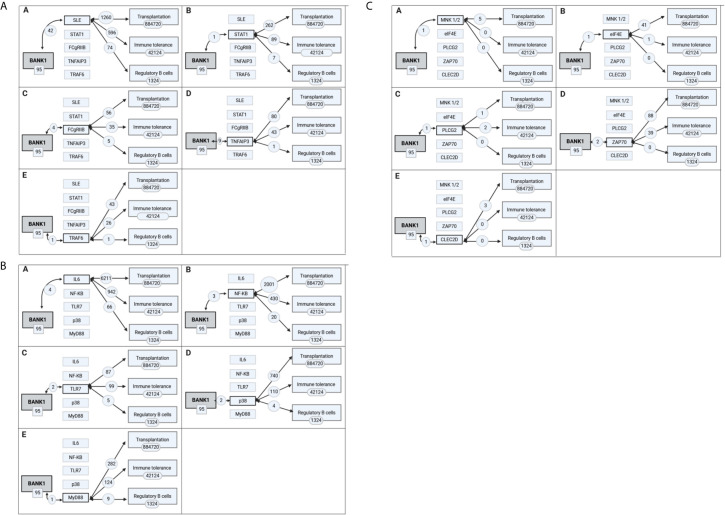
Intermediate biomolecules indirectly linking BANK1 to the concepts of transplantation, immune tolerance and regulatory B cells. The most prevalent biological processes and molecules associated with the BANK1 literature were each used in PubMed searches in combination with transplantation, immune tolerance and regulatory B cells literature. The extent of the overlap with BANK1 literature is shown in **(A)** in A for SLE, in B for STAT1, in C for FCgRIIb, in D for TNFAIP3, and in E for TRAF6; in **(B)** in A for IL6, in B for NF-KB, in C for TLR7, in D for p38, and in E for Myd88; in **(C)** in A for MNK1/2, in B for eIF4E, in C for PLCG2, in D for ZAP70, and in E for CLEC2D.

To specifically explore the link between BANK1 and Bregs, PubMed queries were performed independently between the 14 proteins and autoimmune diseases identified in the previous queries and Bregs (see details of the queries in **Supplementary Table 1**). SLE returned the highest number of PubMed hits with Bregs (74 articles). We used Open Target source, (in “text mining”) to highlight the associations of BANK1 and different diseases in the literature (**Figure 4**). While we focused on search results with occurrences superior to 1 article (in gray in **Table 2**), three proteins (BLK, IRF5 and STAT4) did not have common publications with Bregs and were therefore excluded from further analyses. We extended searches to other proteins of **Table 2**, and identified STAT1, FcγRIIB, TNFAIP3, TRAF6, TLR7, IL6, NF-KB, MyD88, and p38 as having an association with Bregs (**Table 3**). IL6, NF-Kb, p38, and MyD88, four proteins that have pleiotropic roles in the immune response, are present in all immune cells and are involved in most responses to many signals of activation and inflammation but therefore do not allow to find a new role for a molecule whose function is still little known, have been discarded. Since these five other candidate molecules (STAT1, FcγRIIB, TNFAIP3, TRAF6, and TLR7) intersected both BANK1 and Breg in the literature, we performed additional searches to include the terms transplantation, immune regulation, and Bregs. **Figures 5A–C** summarize the results of the PubMed searches and highlight a strong association of these molecules with transplantation, immune tolerance and a more moderate one with Bregs.

**Table 3:** Concepts extracted from the BANK1 literature and the prevalence of each terms with BANK1, or Bregs.

	AND BANK1	AND Bregs
**SLE**	42	74
**BLK**	31	0
**IRF5**	20	0
**STAT4**	20	0
**STAT1**	1	7
**ATG5**	5	0
**ATG7**	1	0
**FcgR2B**	4	5
**PTPN22**	14	0
**PTPN6**	1	0
**TNFAIP3**	9	1
**TNFSF4**	14	0
**TNFRSF14**	1	0
**TRAF6**	1	1
**IL6**	4	66
**NF-kappa B**	3	20
**TLR7**	2	5
**P38**	2	4
**MyD88**	1	9
**MNK1/2**	1	0
**eIF4E**	1	0
**PLCG2**	1	0
**ZAP70**	2	0
**CLEC2D**	1	0

In gray, common occurrences to both searches.

These results indicate a solid link between BANK1 and SLE, in which the role of Bregs in the disease physiopathology has been demonstrated through an association between Bregs defects and disease pathogenesis (19, 20) (**Figure 4**).

### BCR Signaling Links BANK1 and Apoptosis

**Figure 6:**
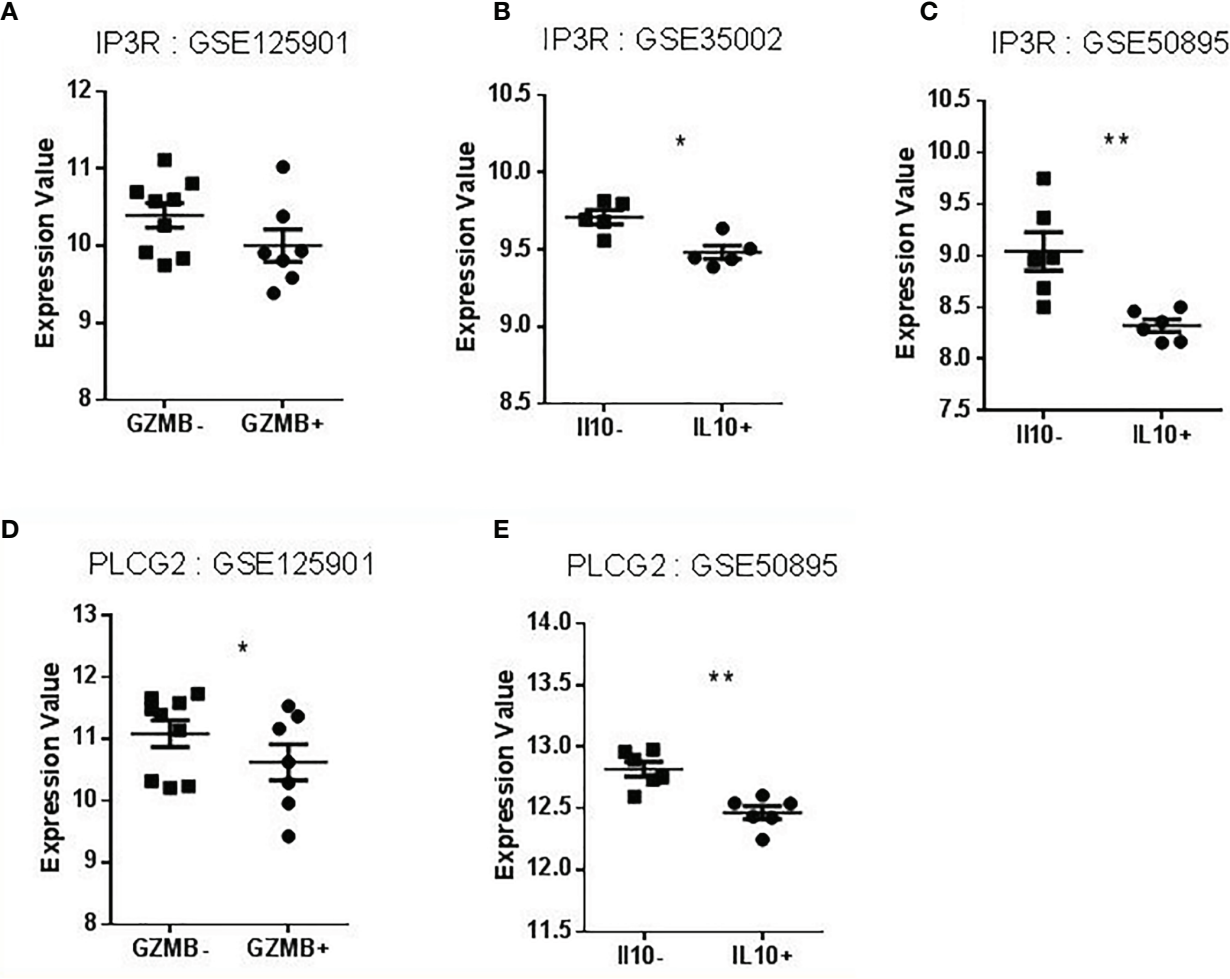
Expression values of IP3R and PLCG2 in datasets selected for validation. **(A–C)** Levels of IP3R expression in B cells compared to Bregs (GZMB^+^ or IL10^+^). **(D–E)** Levels of PLCG2 expression in B cells compared to Bregs (GZMB^+^ or IL10^+^) (*p < 0.05, **p < 0.01).

FcγRIIB, TNFAIP3, TRAF6, and TLR7 are known to play a role in B cell signaling, including BCR and TLR stimulation (21–26). We thus focused our search on BANK1 and BCR signaling. PubMed search between BANK1 AND BCR (BANK1 [tw] OR “B Cell Scaffold Protein with Ankyrin Repeats” [tw] AND BCR [tw]) yielded a mere five publications, in which two articles indicated a role of BANK1 in BCR signaling *via* IP3R (27) and PLCG2 (28). Since IP3R and PLCG2 have prominent role in apoptosis signaling (29–31), we examined the expression of IP3R and PLCG2 in our dataset (GSE125901) and the three public datasets (GSE76272, GSE35002, GSE50895) comparing non-Bregs with Bregs sub-populations (**Figure 6** **)**. Like BANK1, transcript levels of IP3R and PLCG2 were downregulated in Bregs compared to non-Bregs. Based on the gene expression patterns, there may be a potential link between BANK1 and IP3R/PLCG2/Ca^2+^ pathway, Bregs and apoptosis, which requires further investigations, such as phosphorylation status of PLCG2 and IP3R.

### Interaction Network of BANK1: Two GO Linked to Apoptotic Processes

**Figure 7:**
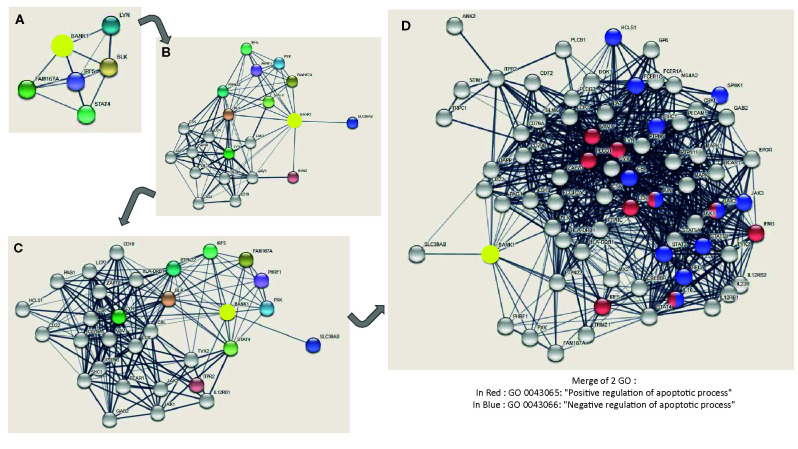
Different levels of BANK1 STRING network and GO related to apoptosis in BANK1 STRING network. The interactions include direct (physical) and indirect (functional) associations. Colored nodes correspond to query proteins and first shell of interactors, and white nodes correspond to second shell of interactors. The thickness of the connection lines indicates the strength of data support (Selected sources: Textmining, Experiments, Database and Co-expression) expressed by edge confidence [from low (0.15) to highest (0.9)]. Minimum required interaction score is based on medium confidence of 0.4. The maximum number of interactions shown are of five interactors for 1^st^ shell and 0 interactor for 2^nd^ shell in **(A)**; of 10 interactors for 1^st^shell and 10 interactors for 2^nd^ shell in **(B)**; of 10 interactors for 1^st^ shell and 20 interactors for 2^nd^ shell in **(C)**; and of 10 interactors for 1^st^ shell and 60 interactors maximum for 2^nd^ shell in **(D)**. In blue, genes involved in GO 0043066: Negative regulation of apoptotic process. In red, genes involved in GO 0043065: Positive regulation of apoptotic process.

To complete the reductionist method (32), we used the STRING approach (https://string-db.org/) (33) to support a link between BANK1 and apoptosis. We displayed different levels of BANK1 STRING network in **Figure 7** to simplify complexity of protein interactions. In the BANK1 network, we found Jak1, a kinase activator of STAT1, one of candidates found in the reductionist approach to be related to BANK1 and Bregs. It is also represented VAV1 and JAK2, two actors of a particular oncogenic apoptotic pathway involving p53, found in tumoral environment (34, 35), but also in TCR signaling, maturation of B cells, and actin polymerization (36) (**Figures 7B–D**). STIM1 and 2, two endoplasmic reticulum Ca^2+^sensors responsible of cytosolic Ca^2+^ regulation are also represented in BANK1 network. These proteins are instrumental for IL10 dependent suppressive properties of IL10^+^ Bregs and control IL10 production in these cells after BCR activation, with PLCG2 and IP3R involvement (37). STIM1 links BANK1 to Bregs regulation. Two GO related to regulation of apoptotic process are linked to BANK1 network: GO 0043066 designated by “Negative regulation of apoptotic process” and GO 0043065 designated by “Positive regulation of apoptotic process” (**Figure 7D**).

### B Cells From Tolerant Patients and Bregs Are More Sensitive to Apoptosis Despite Expressing Opposite BANK1 Expression Patterns

**Figure 8:**
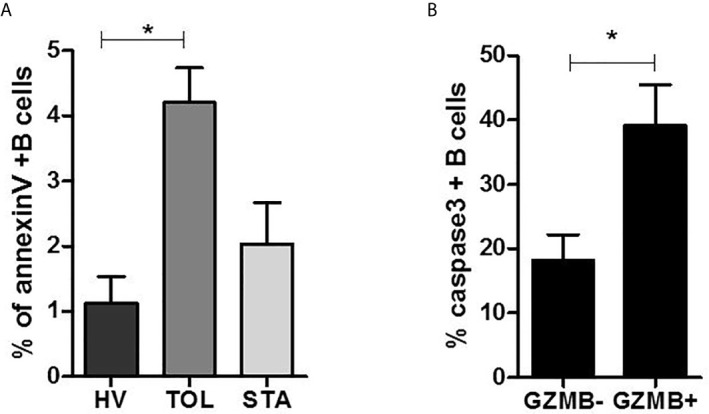
B cells apoptosis in tolerant patients and GZMB^+^ Bregs. **(A)** Percentage of annexin V^+^ B cells in Healthy volunteers (HV), stable patients (STA), and tolerant patients (TOL). **(B)** Percentage of caspase 3^+^ B cells in GZMB^-^ B cells vs. GZMB^+^ Bregs (*p < 0.05).

To experimentally assess the association between BANK1 and apoptosis ex-vivo, we performed caspase 3 (38) and annexin V tests on purified B cells from TOL, HV, and STA patients (**Figure 8A**, unpublished data) and in GZMB^+^ vs GZMB^-^ B cells from HVs [**Figure 8B**, recently published in (10)]. In agreement with our previous studies (5) and literature (39), we observed a parallel increase of BANK1 transcripts in B cells from tolerant patients with a significant increase of apoptotic B cells in TOL compared with HV and STA (1.126 ± 0.8% in HV vs 4.204 ± 1.19% in TOL, p < 0.05) (**Figure 8A** **)**. Interestingly, a significant increase of apoptosis in GZMB^+^ compared with GZMB^-^ B cells was also observed (18.19 ± 8.89% in GZMB^-^ vs 39.18 ± 14.16% in GZMB^+^, p < 0.05) (**Figure 8B** **)** whereas BANK1 expression was reduced in GZMB^+^ Bregs. This observation is in contradiction with published observations, showing similar evolution of BANK1 transcript level and apoptosis, but in accordance with the decrease in GZMB^+^ Bregs, of transcript levels of PLCG2/IP3R (aforementioned pro-apoptotic Ca^2+^ pathway) found to be associated with BANK1 (**Figure 6** **).**

### Expression of Actors of Three Classical Apoptotic Pathways in Bregs

**Figure 9:**
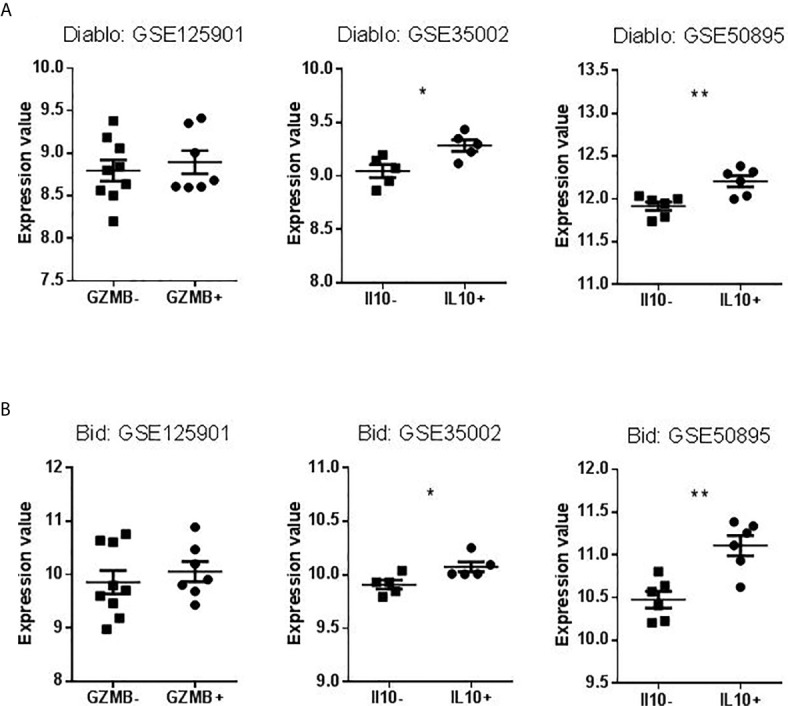
Expression Values of Diablo and Bid in datasets selected for validation. **(A)** Levels of Diablo expression in B cells compared to Bregs (GZMB^+^ or IL10^+^). **(B)** Levels of Bid expression in B cells compared to Bregs (GZMB^+^ or IL10^+^) (*p < 0.05, **p < 0.01).

**Figure 10:**
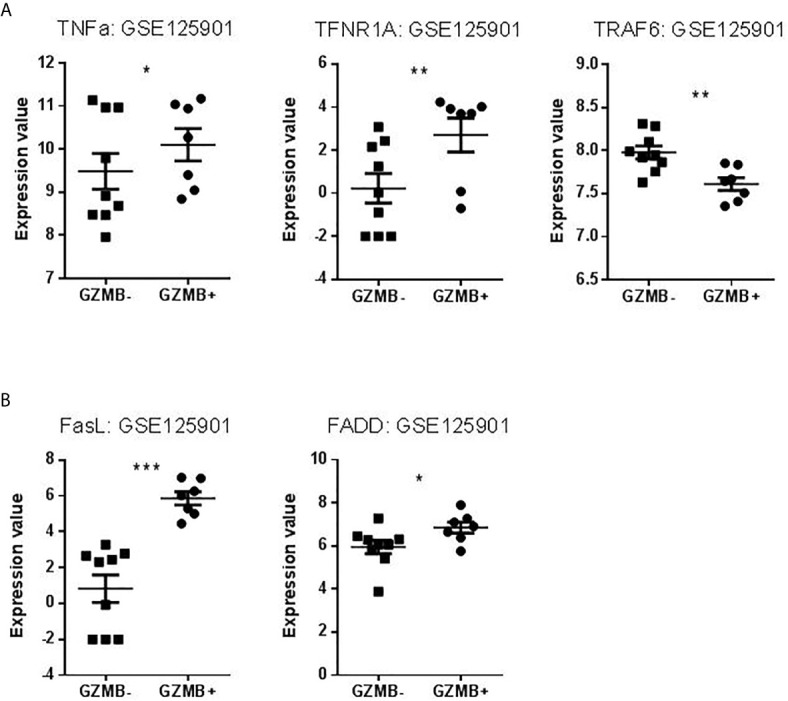
Expression Values of TNF pathway and FASL pathway in our dataset. **(A)** Levels of TNFα, TNFR1A, and TRAF6 expression in GZMB^-^ B cells compared to GZMB^+^ Bregs. **(B)** Levels of FasL and FADD expression in GZMB^-^ B cells compared to GZMB^+^ Bregs (*p < 0.05, **p < 0.01, ***p < 0.001).

We searched to understand which pathway of apoptosis is involved in GZMB^+^ B cells and B cells of tolerant patients that would explain this contradiction. We chose apoptotic actors (based on published apoptosis signaling diagrams), used experimentally (available tools, commercial tests) representative to three classical apoptotic pathways in immunology: intrinsic, FasL, and TNFa pathways. We then checked for the differential expression of these genes in the Bregs data sets. Instrumental mediators of apoptosis such as Diablo and Bid (intrinsic apoptosis way) (**Figures 9A, B** **)**, TNF signaling (represented by TNFα, TNFR1A, and TRAF6) and FasL signaling (represented by FasL and FADD) were however increased in GZMB^+^ Bregs (**Figures 10A, B** **)** compared with GZMB^-^ B cells.

## Discussion

B cells are important players of the immune response known first for their production of antibodies and antigen presentation but also more recently known as instrumental to limit inflammation and preventing auto-immune diseases. These subsets of B cells are called regulatory B cells (Bregs). They have suppressive activities (40–42) on different cells types, T cells, dendritic cells, other B cells and monocytes, namely by promoting regulatory T cells (43). They act through several mechanisms, including IL-10, IL-35, granzyme B (GZMB), TGF-β production, and/or cell-to-cell contact *via* programmed death-ligand 1 (PD-L1), FasL, and glucocorticoid-induced TNFR-related ligand (GITRL) expression (8, 44–46). GZMB^+^ B cells were identified as suppressive B cells involved in growing number of pathological conditions, such as B cell chronic lymphocytic leukemia (47), solid tumor infiltration (48), autoimmune diseases (9) and infections (49). We previously reported that tolerant patients exhibit a higher number of GZMB^+^ B cells that can inhibit effector T cell response by contact and through a GZMB-dependent manner (8) and that BANK1 transcript is upregulated in whole blood after kidney transplantation in tolerant patients (5). We searched for the link between the 2 and to know more about BANK1 role.

BANK1 is an adaptor protein primarily expressed in mature and immature B cells (27). BANK1 lacks predicted enzymatic activity but contains two ankyrin domains, 13 tyrosines susceptible to phosphorylation and interacting with SH2 domains, two proline-rich motifs that interact with SH3 domains, and a Dof-BCAP-BANK motif involved in dimerization (50). BANK1 is a substrate of SYK and is phosphorylated upon BCR activation. Its overexpression in cells triggers a strong BCR-mediated Ca^2+^ mobilization, *via* LYN-mediated phosphorylation of IP3R and intracellular Ca^2+^ release (27). AKT is the major downstream molecule of BANK1 by which BANK1 negatively regulates CD40-mediated B cell activation (51, 52). We published previously that AKT is a leader gene in a rodent model of tolerance in allotransplantation (53) and reported an increase of BANK1 expression in B cells from tolerant patients (5). Inhibition of BANK1 expression by siRNA is associated with a decrease of apoptosis, suggesting that BANK1 display a pro-apoptotic function (39), in accordance, with higher level of apoptosis in B cells from tolerant patients. Finally, BANK1 interacts directly with MyD88 and TRAF6, two major actors of TLR signaling (54) and controls TLR7-mediated type I IFN production and STAT1 activation (55), fitting with the decrease of TLR signaling observed in cells from tolerant patients (56). We found literature associations of FcγRIIb, STAT1, TNFAIP3 and BCR signaling (57–60) and we have previously shown an increased level of FcγRIIb in blood from tolerant recipients (56).

Most of articles on BANK1 report genetic studies showing the importance of BANK1 in autoimmune diseases. Among these diseases, systemic lupus erythematosus (SLE) is the most studied. Thus, functional variants of BANK1 are associated with SLE (52, 54, 61–63), rheumatoid arthritis (64), diffuse cutaneous systemic sclerosis (65, 66), multifocal motor neuropathy (67), type I diabetes (68), autoimmune thyroid disease (69), B cell lymphoma (70), LDL cholesterol (71), psoriasis (72), primary Sjogren’s syndrome (73), polymyositis/dermatomyositis (74), and systemic sclerosis (75), pointing an important functional role for BANK1 in immune system, without, however explaining its role.

In contrast with its over-expression in total B cells from tolerant patients, we observed that BANK1 is decreased in GZMB^+^ Bregs. This is not contradictory with its overexpression in whole blood from tolerant patients regarding the low frequency of GZMB^+^ Bregs in circulation (< 1%) (8). It is thus unlikely, that these GZMB^+^ Bregs contribute to the BANK1 expression observed in such patients. We reported that BANK1 gene expression is not only decreased in GZMB^+^ Bregs, but also in other Breg cell subtypes with different phenotypes and functions. This is concordant with the reduced level of BANK1 observed after B cell activation and in differentiated cells, as tumor B cells (GSE 22529) or plasma cells (GSE 6691), since Breg cells have also been shown to be highly differentiated cells (8, 76–80) and GZMB^+^ Bregs are enriched in plasmablast population (10). Downregulation of BANK1 in these cells could be a hallmark of preplasma phenotype, common to Bregs and plasma cells. On the other hand, it cannot be the hallmark of IL10^+^ Bregs because GZMB^+^ Bregs suppressive activity is not dependent from IL10 and GZMB^+^ Bregs do not produce more IL10 than resting/GZMB^-^ B cell.

Surprisingly, whereas overexpression of BANK1 in B cells from tolerant patients is correlated to higher sensitivity to apoptosis, GZMB^+^ Bregs exhibit an increased apoptotic phenotype despite having lower BANK1 expression. Apoptosis is a physiological phenomenon participating to the homeostasis of its dysregulation may be associated with cancer and/or auto-immune diseases (81, 82). We hypothesize that B cells from tolerant patients, in which BANK1 expression is upregulated and apoptosis increased, may be controlled by phenomenon of homeostatic regulation, favoring certain apoptosis mechanisms. In contrast, GZMB^+^ Bregs harbor lower level of BANK1 with increased suppressive activity suggesting that other mechanisms are involved in this process of regulation. As IP3R and PLCG2 expressions are also decreased, the apoptotic Ca^2+^ pathway seems to be downregulated in GZMB^+^ Bregs. Thanks to this transcriptomic study, we found that other distinct apoptotic pathways, independent from BANK1/IP3R/PLCG2/Ca^2+^, are involved in GZMB^+^ Bregs, as well as in other types of Bregs (CD19^+^CD5^+^ FoxP3^+^ B cells) (83).

Thus, although BANK1 may modulate B cell activation under pathological situations (51, 52), such as by maintaining an inhibitory profile in tolerant patients under activation (5), other apoptotic mechanisms may be involved in Bregs under normal physiological conditions. This is supported by the overexpression of genes in GZMB^+^ Bregs that are instrumental in apoptosis, such as those involved in intrinsic apoptotic, TNF and Fas/FasL pathways (Diablo, Bid, TNFα, TNFR1A, TRAF6, FasL, FADD). The down-regulation of BANK1 in GZMB^+^ Bregs fits with its similar down-regulation in others differentiated cells (18) which are highly prone to apoptosis by mechanisms that are not mediated by CD40 activation, and thus not dependent from BANK1 (84–86).

To note, Aiba et al. found that BCR-mediated calcium mobilization was not significantly changed in BANK1-deficient B cells (51). It was first found that BANK1 was bound to LYN and that the complex formed with PLCG2 then phosphorylated IP3R (27), but later studies finally reported that BLK was the specific partner of BANK1 (28, 62). Lyn may be specific for a protein of Dof-BCAP-BANK family, different from BANK1, playing a redundant role to that of BANK1 in other conditions, since BANK1 is important to prevent runaway of immune response.

**Figure 11:**
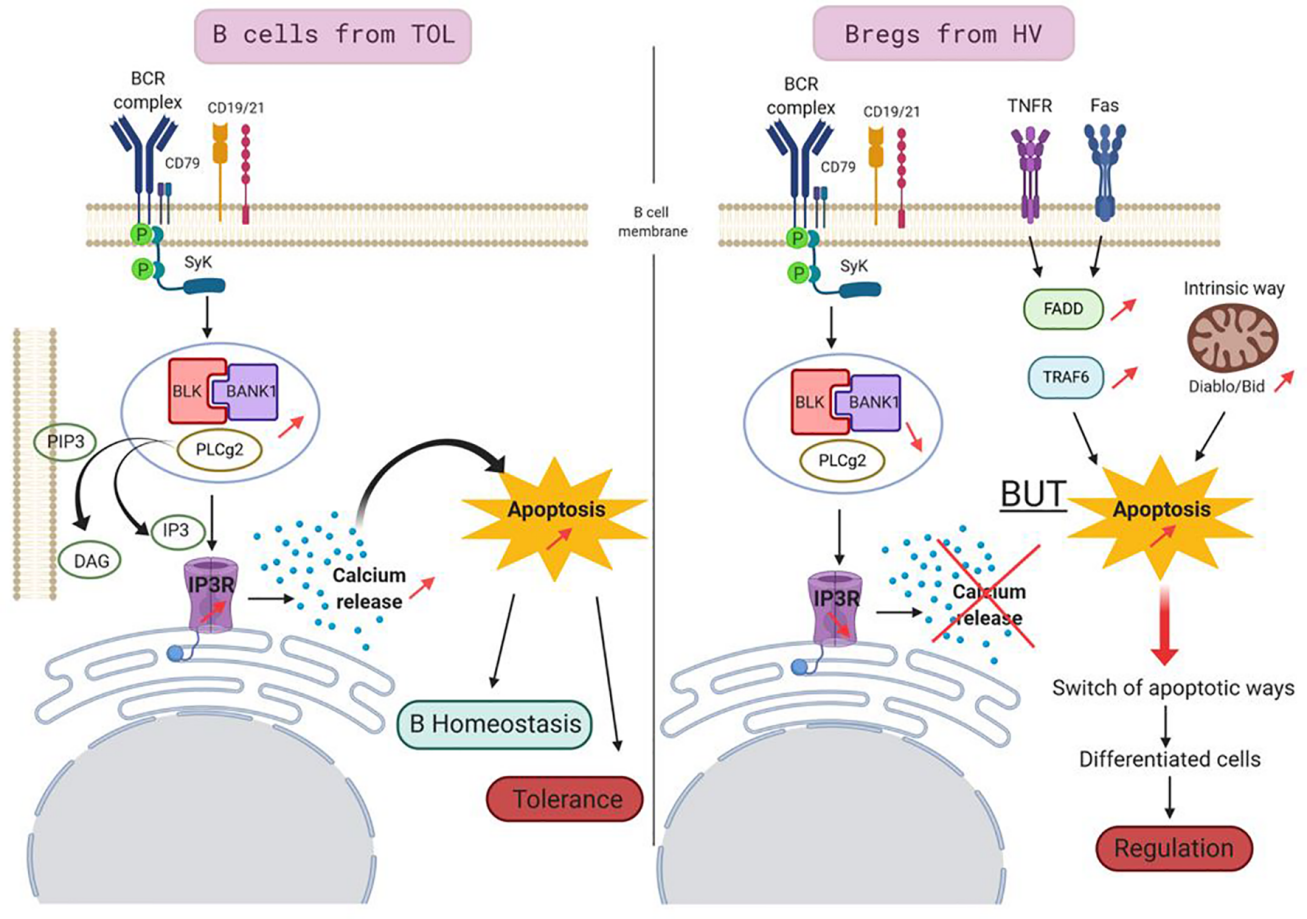
Proposed model for a role of BANK1 in tolerant patients. In B cells of TOL (left panel), BCR stimulation triggers the phosphorylation of adaptor molecule SYK which, then activated, phosphorylates BANK1. BANK1 binds B lymphoid kinase (BLK). This complex interacts with phosphorylated PLCγ2. This new complex is recruited by membrane PIP3s which are cleaved in DAG and IP3. IP3s fixe to IP3 Receptors and trigger intracellular Ca^2+^ release leading to a proapoptotic action. This apoptotic pathway, increased in TOL, could be involved in regulation of homeostasis of these cells and participated to tolerance phenomenon in regulating the survival of potential detrimental cells for the graft. In Bregs from HV (right panel), the BANK1/BLK/PLCγ2 complex is reduced, the intracellular Ca^2+^ is thus not released, this apoptotic pathway is not privileged, but the apoptosis is still increased in the cells. Other apoptotic pathways take over like TNFR/Fas pathways or the intrinsic pathway. This switch of apoptotic pathways in the Bregs may be a feature of differentiated cells, but could also play a role in the suppressive/regulation activity of Bregs.

In conclusion, thanks to our own reports and new experiments as well as publicly available transcriptomic data, we decipher the molecular links between BANK1 and regulatory B cells and we proposed a hypothetical role for BANK1 in tolerance (see Legend of **Figure 11** **).** This article is clearly a basis for future explorations in the fields of Bregs and immune tolerance, and not a complete story that would answers questions of mechanistic processes. Since BANK1 is known to be highly expressed in B cells, especially in naive and memory B cells, a deeper investigation of its role in B cell populations of TOL patients could be assessed in the future.

## Data Availability Statement

Publicly available data sets were analyzed in this study. These data can be found here: https://www.ncbi.nlm.nih.gov/ under the accession numbers: GSE125901, GSE76272, GSE35002, GSE50895, GSE54017, GSE22529, and GSE6691.

## Ethics Statement

Blood samples used in this study were provided to us by the French blood bank (EFS: Etablissement Français du Sang), according to an agreement between our laboratory and this bank. The patients/participants provided their written informed consent to participate in this study.

## Author Contributions

LL wrote the manuscript, performed some figures and final schema, corrected and edited the manuscript, wrote the cover letter, and submitted the article. MC performed apoptosis experiments, some figures and tables and participated in writing and correcting the manuscript. RD participated in the analysis of our omics study. FD participated in the analysis of different omics studies. DC participated in the review of the article. MG participated in the design and interpretation of the study, and in the correction of the manuscript. SB participated in the design and interpretation of the study, in the writing, and in the correction of the manuscript. All authors contributed to the article and approved the submitted version.

## Funding

This work was performed thanks to French government financial support managed by the National Research Agency via the “Investment into the Future” program (ANR-10-IBHU-005), via the LabEX IGO (ANR-11- LABX-0016-01), the ANR project PRELUD (ANR-18-CE17-0019), the ANR project BIKET (ANR-17-CE17-0008), the ANR project KTD-innov (ANR-17-RHUS-0010). RD was supported by a Marie Skłodowska Curie fellowship (IF-EF) from the European Union’s Horizon 2020 Research and Innovation Programme (grant agreement 706296).

## Conflict of Interest

The authors declare that the research was conducted in the absence of any commercial or financial relationships that could be construed as a potential conflict of interest.

## Abbreviations

AI disease, auto immune disease; BANK1, B-cell scaffold with ankyrin repeats 1; Bid, BH3 interacting domain death agonist; BLK, B lymphoid kinase; Bregs, regulatory B cells; CLL, chronic lymphocytic leukemia; CR, chronic rejection; Diablo, direct IAP-binding protein with low PI; FADD, fas associated *via* death domain; FasL, Fas Ligand; FcγRIIB, Fc fragment of IgG receptor IIb; GITR, glucocorticoid-induced TNFR-related ligand; GZMB, granzyme B; HV, healthy volunteers; IP3R, inositol 1,4,5-trisphosphate receptor type 3; IRF5, interferon regulatory factor 5; PD-L1, programmed death-ligand 1; PBMC, peripheral blood mononuclear cells; PLCG2, phospholipase C gamma 2; RA, rheumatoid arthritis; SLE, systemic lupus erythematosus; SSc, systemic sclerosis; STA, patients with long term renal STAble function under classic immunosuppressive treatment; STAT4/1, signal transducer and activator of transcription 4/1; TLR, toll-like receptor; TNFAIP3, tumor necrosis factor alpha-induced protein 3; TNFR1A, tumor necrosis factor receptor superfamily member 1A; TNFα, tumor necrosis factor alpha; TOL, operationally tolerant patients; TRAF6, TNF receptor associated factor 6.
