# Supplementary material for: Connection of BANK1, Tolerance, Regulatory B cells, and Apoptosis: Perspectives of a Reductionist Investigation
Source: Front Immunol. 2021 Mar 18;12:589786. doi: 10.3389/fimmu.2021.589786 (PMC8015775; doi:10.3389/fimmu.2021.589786)
Supplement: Supplementary file 1 [file Table_1.pdf]

	AND BANK1	AND Bregs
**SLE**	(BANK1[tw] OR BANK-1[tw] OR "B Cell Scaffold Protein With Ankyrin Repeats"[tw]) AND ("Systemic lupus erythematosus"[tw] OR "SLE"[tw])	(Breg[tw] OR "regulatory B cells"[tw] OR "regulatory B lymphocyte"[tw] OR "regulatory B cells"[tw]) AND ("Systemic lupus erythematosus"[tw] OR "SLE"[tw])
**BLK**	(BANK1[tw] OR BANK-1[tw] OR "B Cell Scaffold Protein With Ankyrin Repeats"[tw]) AND (BLK[tw] OR "BLK Proto-Oncogene, Src Family Tyrosine Kinase "[tw] OR "B Lymphoid Tyrosine Kinase[tw])	(Breg[tw] OR "regulatory B cells"[tw] OR "regulatory B lymphocyte"[tw] OR "regulatory B cells"[tw])AND (BLK[tw] OR "BLK Proto-Oncogene, Src Family Tyrosine Kinase "[tw] OR "B Lymphoid Tyrosine Kinase[tw])
**IRF5**	(BANK1[tw] OR BANK-1[tw] OR "B Cell Scaffold Protein With Ankyrin Repeats"[tw]) AND (IRF5[tw] OR IRF-5[tw] OR "Interferon Regulatory Factor 5"[tw] OR SLEB10[tw])	(Breg[tw] OR "regulatory B cells"[tw] OR "regulatory B lymphocyte"[tw] OR "regulatory B cells"[tw]) AND (IRF5[tw] OR IRF-5[tw] OR "Interferon Regulatory Factor 5"[tw] OR SLEB10[tw])
**STAT4**	(BANK1[tw] OR BANK-1[tw] OR "B Cell Scaffold Protein With Ankyrin Repeats"[tw]) AND (STAT4[tw] OR "Signal Transducer And Activator Of Transcription 4"[tw] OR SLEB11[tw])	(Breg[tw] OR "regulatory B cells"[tw] OR "regulatory B lymphocyte"[tw] OR "regulatory B cells"[tw]) AND (STAT4[tw] OR "Signal Transducer And Activator Of Transcription 4"[tw] OR SLEB11[tw])
**STAT1**	(BANK1[tw] OR BANK-1[tw] OR "B Cell Scaffold Protein With Ankyrin Repeats"[tw]) AND (STAT1[tw] OR "Signal Transducer And Activator Of Transcription 1"[tw])	(Breg[tw] OR "regulatory B cells"[tw] OR "regulatory B lymphocyte"[tw] OR "regulatory B cells"[tw]) AND (STAT1[tw] OR "Signal Transducer And Activator Of Transcription 1"[tw])
**ATG5**	(BANK1[tw] OR BANK-1[tw] OR "B Cell Scaffold Protein With Ankyrin Repeats"[tw]) AND (ATG5[tw] OR ATG-5[tw]OR ATGR[tw] OR "Autophagy Related 5" [tw])	(Breg[tw] OR "regulatory B cells"[tw] OR "regulatory B lymphocyte"[tw] OR "regulatory B cells"[tw]) AND (ATG5[tw] OR ATG-5[tw]OR ATGR[tw] OR "Autophagy Related 5" [tw])
**ATG7**	(BANK1[tw] OR BANK-1[tw] OR "B Cell Scaffold Protein With Ankyrin Repeats"[tw]) AND (ATG7[tw] OR ATG-7[tw] OR "Autophagy Related 7"[tw])	(Breg[tw] OR "regulatory B cells"[tw] OR "regulatory B lymphocyte"[tw] OR "regulatory B cells"[tw]) AND (ATG7[tw] OR ATG-7[tw] OR "Autophagy Related 7"[tw])
**FcgR2B**	(BANK1[tw] OR BANK-1[tw] OR "B Cell Scaffold Protein With Ankyrin Repeats"[tw]) AND (FCGR2B[tw] OR "Fc Fragment Of IgG Receptor IIb "[tw] OR "Fc Gamma Receptor IIb"[tw] OR Fc-Gamma RII-B [tw] OR FcRII-B[tw] OR CDw32[tw] OR IGFR2[tw] OR CD32[tw])	(Breg[tw] OR "regulatory B cells"[tw] OR "regulatory B lymphocyte"[tw] OR "regulatory B cells"[tw]) AND (FCGR2B[tw] OR "Fc Fragment Of IgG Receptor IIb "[tw] OR "Fc Gamma Receptor IIb"[tw] OR Fc-Gamma RII-B [tw] OR FcRII-B[tw] OR CDw32[tw] OR IGFR2[tw] OR CD32[tw])
**PTPN22**	(BANK1[tw] OR BANK-1[tw] OR "B Cell Scaffold Protein With Ankyrin Repeats"[tw]) AND (PTPN22[tw] OR "Protein Tyrosine Phosphatase Non-Receptor Type 22"[tw] OR PTPN8[tw])	(Breg[tw] OR "regulatory B cells"[tw] OR "regulatory B lymphocyte"[tw] OR "regulatory B cells"[tw]) AND (PTPN22[tw] OR "Protein Tyrosine Phosphatase Non-Receptor Type 22"[tw] OR PTPN8[tw])
**PTPN6**	(BANK1[tw] OR BANK-1[tw] OR "B Cell Scaffold Protein With Ankyrin Repeats"[tw]) AND (PTPN6[tw] OR "Protein Tyrosine Phosphatase Non-Receptor Type 6"[tw])	(Breg[tw] OR "regulatory B cells"[tw] OR "regulatory B lymphocyte"[tw] OR "regulatory B cells"[tw]) AND (PTPN6[tw] OR "Protein Tyrosine Phosphatase Non-Receptor Type 6"[tw])
**TNFAIP3**	(BANK1[tw] OR BANK-1[tw] OR "B Cell Scaffold Protein With Ankyrin Repeats"[tw]) AND (TNFAIP3[tw] OR "Protein Tyrosine Phosphatase Non-Receptor Type 6"[tw] OR "Tumor Necrosis Factor Alpha-Induced Protein 3"[tw] OR "Tumor Necrosis Factor Inducible Protein A20"[tw] OR TNFA1P2[tw])	(Breg[tw] OR "regulatory B cells"[tw] OR "regulatory B lymphocyte"[tw] OR "regulatory B cells"[tw]) AND (TNFAIP3[tw] OR "Protein Tyrosine Phosphatase Non-Receptor Type 6"[tw] OR "Tumor Necrosis Factor Alpha-Induced Protein 3"[tw] OR "Tumor Necrosis Factor Inducible Protein A20"[tw] OR TNFA1P2[tw])
**TNFSF4**	(BANK1[tw] OR BANK-1[tw] OR "B Cell Scaffold Protein With Ankyrin Repeats"[tw]) AND (TNFSF4 [tw] OR "TNF Superfamily Member 4"(tw] OR "Tumor Necrosis Factor Ligand Superfamily Member 4"[tw] OR OX-40L(tw] OR CD134L[tw] OR CD252[tw] OR OX40L[tw])	(Breg[tw] OR "regulatory B cells"[tw] OR "regulatory B lymphocyte"[tw] OR "regulatory B cells"[tw]) AND (TNFSF4 [tw] OR "TNF Superfamily Member 4"(tw] OR "Tumor Necrosis Factor Ligand Superfamily Member 4"[tw] OR OX-40L(tw] OR CD134L[tw] OR CD252[tw] OR OX40L[tw])
**TNFRSF14**	(BANK1[tw] OR BANK-1[tw] OR "B Cell Scaffold Protein With Ankyrin Repeats"[tw]) AND (TNFRSF14[tw] OR "TNF Receptor Superfamily Member 14"[tw] OR "Tumor Necrosis Factor Receptor Superfamily Member 14"[tw] OR CD270[tw] OR "CD40-Like Protein"[tw])	(Breg[tw] OR "regulatory B cells"[tw] OR "regulatory B lymphocyte"[tw] OR "regulatory B cells"[tw]) AND (TNFRSF14[tw] OR "TNF Receptor Superfamily Member 14"[tw] OR "Tumor Necrosis Factor Receptor Superfamily Member 14"[tw] OR CD270[tw] OR "CD40-Like Protein"[tw])
**TRAF6**	(BANK1[tw] OR BANK-1[tw] OR "B Cell Scaffold Protein With Ankyrin Repeats"[tw]) AND (TRAF6[tw] OR TRAF-6[tw] OR "TNF Receptor Associated Factor 6"[tw])	(Breg[tw] OR "regulatory B cells"[tw] OR "regulatory B lymphocyte"[tw] OR "regulatory B cells"[tw]) AND (TRAF6[tw] OR TRAF-6[tw] OR "TNF Receptor Associated Factor 6"[tw])
**IL6**	(BANK1[tw] OR BANK-1[tw] OR "B Cell Scaffold Protein With Ankyrin Repeats 1") AND ("IL6"[tw] OR "Interleukin 6"[tw])	("Bregs"[tw] OR "regulatory B cells"[tw] OR "B reg"[tw] OR "Breg"[tw]) AND ("IL6"[tw] OR "Interleukin 6"[tw] OR "IFNB2"[tw] OR "BSF2"[tw] OR "HGF"[tw])
**NF-kappa B**	(BANK1[tw] OR BANK-1[tw] OR "B Cell Scaffold Protein With Ankyrin Repeats 1") AND ("NF-KappaB"[tw] OR "NFKB"[tw] OR "Nuclear Factor Kappa B"[tw])	("Bregs"[tw] OR "regulatory B cells"[tw] OR "B reg"[tw] OR "Breg"[tw]) AND ("NF-KappaB"[tw] OR "NFKB"[tw] OR "Nuclear Factor Kappa B"[tw])
**TLR7**	(BANK1[tw] OR BANK-1[tw] OR "B Cell Scaffold Protein With Ankyrin Repeats 1") AND ("TLR7"[tw] OR "Toll like receptor 7"[tw] OR "TLR7-like"[tw])	("Bregs"[tw] OR "regulatory B cells"[tw] OR "B reg"[tw] OR "Breg"[tw]) AND ("TLR7"[tw] OR "Toll like receptor 7"[tw] OR "TLR7-like"[tw])
**P38**	(BANK1[tw] OR BANK-1[tw] OR "B Cell Scaffold Protein With Ankyrin Repeats 1") AND ("p38"[tw] OR "Mitogen-Activated Protein Kinase 14"[tw] OR "MAP Kinase 14"[tw] OR "P38 MAP Kinase"[tw])	("Bregs"[tw] OR "regulatory B cells"[tw] OR "B reg"[tw] OR "Breg"[tw]) AND ("p38"[tw] OR "Mitogen-Activated Protein Kinase 14"[tw] OR "MAP Kinase 14"[tw] OR "P38 MAP Kinase"[tw])
**MyD88**	(BANK1[tw] OR BANK-1[tw] OR "B Cell Scaffold Protein With Ankyrin Repeats 1") AND ("MyD88"[tw] OR "MyD88D"[tw] OR "Myeloid Differentiation Primary Response 88"[tw] OR "MYD88 Innate Immune Signal Transduction Adaptor"[tw])	("Bregs"[tw] OR "regulatory B cells"[tw] OR "B reg"[tw] OR "Breg"[tw]) AND ("MyD88"[tw] OR "MyD88D"[tw] OR "Myeloid Differentiation Primary Response 88"[tw] OR "MYD88 Innate Immune Signal Transduction Adaptor"[tw])
**MNK1/2**	(BANK1[tw] OR BANK-1[tw] OR "B Cell Scaffold Protein With Ankyrin Repeats 1") AND ("MNK1"[tw] OR "MNK2"[tw] OR "MAPK Interacting Serine/Threonine Kinase 1"[tw] OR "MAPK Interacting Serine/Threonine Kinase 2"[tw])	("Bregs"[tw] OR "regulatory B cells"[tw] OR "B reg"[tw] OR "Breg"[tw]) AND ("MNK1"[tw] OR "MNK2"[tw] OR "MAPK Interacting Serine/Threonine Kinase 1"[tw] OR "MAPK Interacting Serine/Threonine Kinase 2"[tw])
**eIF4E**	(BANK1[tw] OR BANK-1[tw] OR "B Cell Scaffold Protein With Ankyrin Repeats 1") AND ("eIF4E"[tw] OR "E74 Like ETS Transcription Factor 4"[tw])	("Bregs"[tw] OR "regulatory B cells"[tw] OR "B reg"[tw] OR "Breg"[tw]) AND ("eIF4E"[tw] OR "E74 Like ETS Transcription Factor 4"[tw])
**PLCG2**	(BANK1[tw] OR BANK-1[tw] OR "B Cell Scaffold Protein With Ankyrin Repeats 1") AND ("PLCG2"[tw] OR "Phospholipase C Gamma 2"[tw] OR "PLC-Gamma-2"[tw])	("Bregs"[tw] OR "regulatory B cells"[tw] OR "B reg"[tw] OR "Breg"[tw]) AND ("PLCG2"[tw] OR "Phospholipase C Gamma 2"[tw] OR "PLC-Gamma-2"[tw])
**ZAP70**	(BANK1[tw] OR BANK-1[tw] OR "B Cell Scaffold Protein With Ankyrin Repeats 1") AND ("Zeta Chain Of T Cell Receptor Associated Protein Kinase 70 "[tw] OR "TZK"[tw] OR "ZAP70"[tw] OR " STCD"[tw] OR "Zeta-Chain (TCR) Associated Protein Kinase 70kDa"[tw])	("Bregs"[tw] OR "regulatory B cells"[tw] OR "B reg"[tw] OR "Breg"[tw]) AND ("Zeta Chain Of T Cell Receptor Associated Protein Kinase 70 "[tw] OR "TZK"[tw] OR "ZAP70"[tw] OR " STCD"[tw] OR "Zeta-Chain (TCR) Associated Protein Kinase 70kDa"[tw])
**CLEC2D**	(BANK1[tw] OR BANK-1[tw] OR "B Cell Scaffold Protein With Ankyrin Repeats 1") AND ("CLEC2D"[tw] OR "C-Type Lectin Domain Family 2 Member D "[tw] OR "CLAX"[tw] OR "LLT1"[tw])	("Bregs"[tw] OR "regulatory B cells"[tw] OR "B reg"[tw] OR "Breg"[tw]) AND ("CLEC2D"[tw] OR "C-Type Lectin Domain Family 2 Member D "[tw] OR "CLAX"[tw] OR "LLT1"[tw])

Supplementary Table
